# Investigation of discordant SARS-CoV-2 RT-PCR results using minimally processed saliva

**DOI:** 10.1038/s41598-022-06642-5

**Published:** 2022-02-18

**Authors:** Dawn White, Jimmy Gu, Catherine-Jean Steinberg, Deborah Yamamura, Bruno J. Salena, Cynthia Balion, Carlos D. M. Filipe, Alfredo Capretta, Yingfu Li, John D. Brennan

**Affiliations:** 1 Biointerfaces Institute, McMaster University, Hamilton, ON L8S 4L8 Canada; 2 Department of Biochemistry and Biomedical Sciences, McMaster University, Hamilton, ON L8N 3Z5 Canada; 3 Department of Medicine, McMaster University, Hamilton, ON L8N 3Z5 Canada; 4 Michael DeGroote Institute of Infectious Disease Research, McMaster University, Hamilton, ON L8N 3Z5 Canada; 5 Department of Pathology and Molecular Medicine, McMaster University, Hamilton, ON L8N 3Z5 Canada; 6 Department of Chemical Engineering, McMaster University, Hamilton, ON L8S 4M1 Canada; 7 School of Biomedical Engineering, McMaster University, Hamilton, ON L8S 4K1 Canada

**Keywords:** Viral infection, Bioanalytical chemistry

## Abstract

Saliva is an attractive sample for coronavirus disease 2019 testing due its ease of collection and amenability to detect viral RNA with minimal processing. Using a direct-to-RT-PCR method with saliva self-collected from confirmed COVID-19 positive volunteers, we observed 32% false negative results. Confirmed negative and healthy volunteer samples spiked with 10^6^ genome copies/mL of heat-inactivated severe acute respiratory syndrome coronavirus 2 showed false negative results of 10% and 13%, respectively. Additional sample heating or dilution of the false negative samples conferred only modest improvements. These results highlight the potential to significantly underdiagnose COVID-19 infections when testing directly from minimally processed heterogeneous saliva samples.

## Introduction

Severe acute respiratory syndrome coronavirus 2 (SARS-CoV-2) has impacted daily life on a global scale since its emergence in December 2019. Scientists rallied quickly to gather and disseminate information to create sensitive molecular detection methods for SARS-CoV-2 to protect the population^1^. Public health authorities have relied heavily on the “gold standard” reverse-transcription polymerase chain reaction (RT-PCR) testing of nasopharyngeal swabs (NPS). However, this type of sample collection requires professional healthcare workers for procurement, which has prompted researchers to evaluate saliva as a simple, self-collectable specimen. Many studies have shown comparable diagnostic sensitivity for viral genomic RNA extracted from saliva specimens versus NPS when using RT-PCR for COVID-19 detection^2^. To further increase simplicity, several methods, such as the covidSHIELD test developed at the University of Illinois Urbana-Champaign (UIUC)^3^, the SalivaDirect test developed at Yale^4^ and the SalivaSTAT method developed at the Medical College of Georgia^5^ utilize direct analysis from unprocessed saliva to avoid the RNA extraction step.

Our groups are actively involved in the development of a point-of-care antigen test for COVID-19 utilizing minimally processed saliva, which is simply allowed to settle for 5 min followed by dilution of the supernatant by 50% with a buffer, as the specimen^6^. We chose the covidSHIELD RT-PCR processing method to aid in validation of our test due to its relative ease and reported sensitivity. However, it was observed during test development that the inherent complexity of saliva^7^ produced a significant number of false negative SARS-CoV-2 RT-PCR results, prompting us to investigate further.

## Methods

### Assessment center and healthy volunteer saliva samples

The research and all associated experimental protocols, including those related to sample collection, processing, and testing were approved by the Research Ethics Board of McMaster University and its affiliated hospitals (Hamilton Health Sciences and St. Joseph's Healthcare). The research was performed in accordance with the institutional guidelines/regulations and the Declaration of Helsinki. Informed consent was obtained from all participants (≥ 18 years of age) and/or their legal guardians attending a Hamilton Health Sciences affiliated COVID-19 assessment center (Hamilton, Ontario) for NPS SARS-CoV-2 testing. Saliva (1 to 5 mL) was self-collected under supervision following the instructions accompanying a provided saliva collection kit immediately after the nasopharyngeal swab. Patients were confirmed as SARS-CoV-2 positive (22 patients: NPS positive) or negative (92 patients: NPS negative) from the NPS sample at the Hamilton Regional Laboratory Medicine Program at St. Joseph’s Healthcare (Hamilton) using an established method^8^. The patient saliva samples, which were collected off-campus, were stored at 4 °C for < 72 h prior to transport to McMaster University for immediate storage at −80 °C. Healthy adult volunteers (23) from the McMaster University research community provided saliva samples in sterile 50 mL polypropylene tubes following the same self-collection instructions; these samples were aliquoted then frozen or used on the same day as collection.

### RT-PCR

RT-PCR was performed using the Power SYBR® Green RNA-to-C_T_™ 1-step Kit (Fisher Scientific, Ottawa, Ontario, Canada) with the CDC N1 and RNase P forward and reverse primers from Integrated DNA Technologies (IDT: NC, USA). All assessment center and healthy volunteer saliva samples were processed based on the original covidSHIELD method described by Ranoa et al.^3^. Briefly, a 20–50 µL aliquot of the saliva sample was heated at 95 °C for 30 min followed by the addition of an equal volume of 2 × TBE-Tw buffer (100 mM Tris pH 7.5, 90 mM boric acid, 1 mM EDTA, 1% Tween-20). Saliva samples were then incubated at room temperature for 5 min to allow any natural salivary particulates to settle to the bottom of the tube. The sample supernatant (5 µL) was mixed with 15 µL of RT-PCR master mix (prepared as instructed) containing 200 nM of N1 or RNase P forward and reverse primers; samples were run on a Biorad CFX96 Touch Real Time PCR system (Hercules, CA, USA). The cycling parameters were: RT—48 °C, 30 min; PCR—95 °C, 10 min, followed by 40 cycles of 95 °C for 15 s then 60 °C for 1 min; melt curve—95 °C for 15 s, 60 °C for 15 s, 95 °C for 15 s. Based on calibration curves (Supplementary Fig. S1) and the limit of detection obtained with these reaction conditions (see details in Supplementary), a threshold cycle value (C_t_ value) of > 36 was considered negative.

### Spiking saliva samples with heat-inactivated SARS-CoV-2; heating and diluting saliva samples

Heat-inactivated 2019 Novel Coronavirus (Isolate USA-WA/2020) was purchased from the ATCC through Cedarlane (Burlington, Ontario, Canada). SARS-CoV-2 NPS negative and healthy volunteer saliva samples were spiked with 4.2 × 10^6^ genome copies (gc)/mL of heat-inactivated SARS-CoV-2 prior to processing for RT-PCR as described above. Saliva samples that were heated prior to spiking and RT-PCR processing were incubated at 95 °C for 30 min. Saliva samples that were diluted prior to RT-PCR processing were done so with an equal volume of sterile RNase-free water.

## Results

Prior to assessing saliva samples, calibration curves were generated for three common SARS-CoV-2 genes (N1, E, and RdRp) using either transcribed or extracted viral RNA to create standard curves. However, when SARS-CoV-2-spiked negative saliva samples were tested, amplification with both the E and RdRp primers failed while amplification with the N1 primers produced the expected result, as did amplification of the RNase P gene. Hence, only the N1 gene was tested going forward. We note that Vogels et al.^4^ reported a nearly identical observation, in that only the N1 gene, and not the N2, E, or orf1 genes, worked with their SalivaDirect assay.

All saliva samples included in this study gave a positive result for the human RNase P internal control using the processing method of the covidSHIELD RT-PCR laboratory developed test^3^ (Supplementary Table S1). The time between off-site saliva sample collection, and receipt and freezing at the university laboratory (< 72 h) posed no concern with respect to sample integrity. In addition, we did not observe an appreciable difference in RT-PCR C_t_ values when the same saliva sample was tested after a freeze–thaw cycle (see Tables S2 and S3). These observations are in agreement with results reported by Herrera et al*.*^9^ and Ott et al.^10^ who have reported that saliva samples are stable for up to 15 days at 4 °C^9^ and unaffected by a single freeze–thaw cycle^10^. A trial experiment that included Proteinase K as a protective agent against RNA degradation from salivary RNases, as described in Vogels et al.,^4^ resulted in failed RT-PCR amplification and therefore was not included in the saliva processing protocol (see details in Supplementary Information).

**Figure 1:**
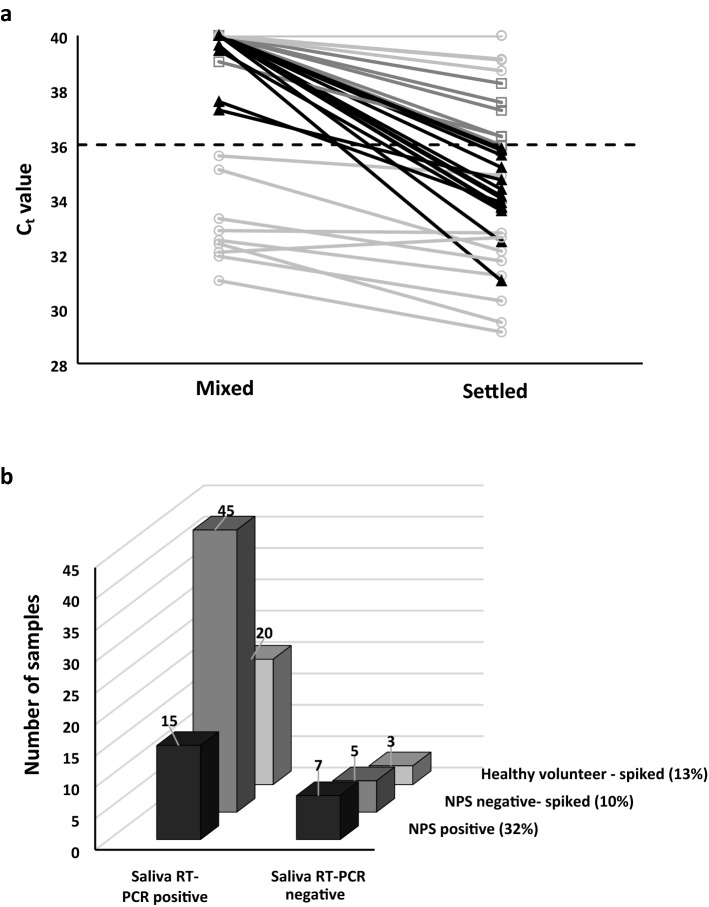
Detection of SARS-CoV-2 in minimally processed saliva samples from COVID-19 positive and negative volunteers. (**a**) Improved detection of spiked saliva samples allowed to settle after processing versus being mixed after processing. Solid black triangles represent samples that became detectable (C_t_ < 36) after settling; dark grey open squares represent samples that showed improved C_t_ values but remained above the cut-off C_t_ value (C_t_ > 36, black dashed line); light grey open circles represent samples that were either detectable in both groups or did not show an appreciable change in C_t_ value. A C_t_ value of 40 represents a negative result. (**b**) Prevalence of false negative SARS-CoV-2 RT-PCR outcomes using minimally processed saliva samples. The sample number is indicated above the data columns. The percent of false negatives is shown in brackets beside each group. All NPS positive saliva samples were tested as is. All NPS negative and healthy volunteer saliva samples were spiked with 10^6^ genome copies/mL heat-inactivated SARS-CoV-2 prior to testing. All samples tested at least in triplicate. See Supplementary Fig. S2 for individual sample C_t_ values.

To test our workflow and reagents with the covidSHIELD RT-PCR processing method, we initially examined SARS-CoV-2 negative saliva samples (NPS negative) spiked with 10^6^ gc/mL heat-inactivated SARS-CoV-2. At this “viral load”, a C_t_ value of 29 was expected based on our calibration data (Supplementary Fig. S1). We observed a significant number of RT-PCR negatives (C_t_ value > 36) with the spiked samples when the processed saliva was mixed prior to addition to the amplification mixture (Fig.  1a). Allowing the same samples to settle improved detection markedly, therefore this settling step was included in all subsequent experiments.

All assessment center saliva samples that tested SARS-CoV-2 negative by NPS also tested negative using the RT-PCR method (100% specificity). However, only 15 of the 22 SARS-CoV-2 that were positive for NPS samples also showed a positive result when using the RT-PCR method with the paired saliva sample (68% sensitivity, see Fig. 1b: NPS positive), with an average C_t_ value of 31.7 ± 2.5 and C_t_ range of 26.6–34.3 (Supplementary Fig. S2). We noticed no difference in the rate of RT-PCR success or failure between the wildtype (9 samples) Alpha (10 samples) and Gamma (3 samples) variants that were part of the 22 COVID-19 positive saliva samples (Figure S2). Note that the Delta and Omicron variants were not in circulation in Ontario during the period of patient sample collection. Given that SARS-CoV-2 mutations primarily affect the gene for the Spike protein, rather than the Nucleocapsid protein, amplification of the N1 gene was not expected to be impacted by the presence of variants.

 The poor solubility led us to question the effect of saliva sample quality (viscosity, particulates) on the test. Physical examination of all the study samples revealed varying viscosity (ranging from water-like to highly viscous with a heterogeneous composition), clarity (clear, cloudy, turbid) and degree of particulate matter (< 10% to > 75% of the sample volume). We evaluated 50 representative SARS-CoV-2 negative saliva samples—from clear and watery to turbid and viscous—by spiking each with 10^6^ gc/mL heat-inactivated SARS-CoV-2. Despite having identical “viral loads”, these samples showed significant variability in the C_t_ values (average C_t_ value of 31.0 ± 2.2; C_t_ range of 27.4–34.9); more concerning was that five samples showed a negative RT-PCR result (Fig. 1b: NPS negative; Supplementary Fig. S2) that did not correlate with the physical quality of the sample, suggesting the presence of chemical components that interfered with the assay.

We then questioned whether saliva samples collected from assessment center volunteers, who presented with symptoms of SARS-CoV-2 infection or suspected exposure to the virus, caused greater interference than samples from healthy people. A set of 23 healthy volunteer saliva samples were spiked and analyzed as described above. A similar result was obtained wherein three of the samples produced a negative RT-PCR result even after spiking (Fig. 1b: Healthy volunteer; Supplementary Fig. S2) (average C_t_ value of 31.5 ± 1.7; C_t_ range of 28.5–34.1).

**Figure 2:**
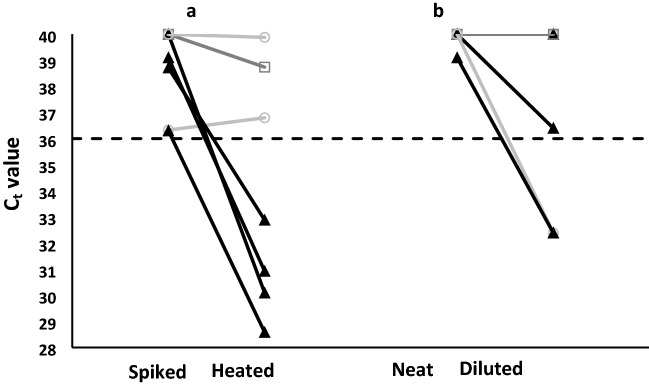
Effect of heat treatment or dilution of spiked saliva samples prior to testing. (**a**) Saliva samples from NPS negative or healthy volunteers that produced a false negative result after spiking with 10^6^ genome copies/mL heat-inactivated SARS-CoV-2 (Spiked) compared with the same saliva samples heated prior to spiking (Heated). (**b**) Spiked saliva samples that produced a false negative result (Neat) tested after dilution (Diluted). Solid black triangles represent samples that became detectable (C_t_ < 36) after settling; dark grey open squares represent samples that showed improved C_t_ values but remained above the cut-off C_t_ value (C_t_ > 36, black dashed line); light grey open circles represent samples that were either detectable in both groups or did not show an appreciable change in C_t_ value (In panel B only, the light grey open circle represents 3 identical, undetectable samples.) A C_t_ value of 40 represents a negative result; the black dashed line at C_t_ = 36 represents the cut-off value for our system. Samples tested at least in duplicate.

Saliva is a complex matrix reported to contain unknown RT-PCR inhibitors^11^ and RNases that could degrade viral RNA upon release from the capsid^12^. To assess if such inhibitors contributed to the observed false negative results with the spiked saliva samples, four SARS-CoV-2 negative and three healthy volunteer saliva samples that did not respond to spiking were heated at 95 °C for 30 min to denature/degrade salivary interferants prior to spiking and processing as before. Four of the seven samples showed positive C_t_ values (average ΔC_t_ [spiked-heated] of 7.9 ± 1.7; C_t_ range of 28.6–32.9) (Fig. 2a).

To determine if chemical interferants could be sufficiently reduced by dilution, which is often used to reduce viscosity^13^ and aid with automated sample processing^14^, saliva samples were diluted 50% before the 50% dilution step incorporated in the RT-PCR processing method creating a 25% saliva solution prior to addition to the RT-PCR reaction. The saliva samples chosen for examination were four SARS-CoV-2 positive samples that tested negative using the RT-PCR method and two healthy volunteer samples whose saliva remained negative after spiking with SARS-CoV-2. The SARS-CoV-2 positive samples remained negative after dilution while the two healthy volunteer samples gave positive results, with a reduction in C_t_ value of 7.6 and 6.7 units (Fig. 2b). This variable drop in C_t_ value with saliva dilution was also observed when the human control RNase P was the RT-PCR target, which in this case was evaluated using a ten-fold dilution to further reduce potential interferants (see Table S4).

## Discussion

Accurate reporting of COVID-19 infections to monitor populations and control outbreaks has been a primary goal worldwide from the start of the pandemic. Among the various methods currently in use, tests using saliva have the advantage of easy sample acquisition, which could allow collection without a health-care practitioner. However, our study has indicated that minimally processed saliva poses a problem for conventional RT-PCR assays due to complications surrounding its physical attributes (viscosity, heterogeneity) and unidentified chemical constituents.

A key observation was that dispersal of saliva particulates via sample mixing prior to the RT-PCR assay increased the number of observed false negative results and increased C_t_ values generally, even in positive samples (Fig. 1a). Allowing the prepared sample to settle for at least 5 min after processing greatly improved RT-PCR detectability. In our hands, brief centrifugation worked as well as settling (Supplementary Fig. S3), although Ranoa et al.^3^ did not observe the same benefit. Based on the visual assessment of several hundred assessment center negative saliva samples including and beyond those used for this study, there was no obvious correlation between any physical attribute and an erroneous RT-PCR result. Furthermore, the rate of false negative results did not seem to depend on the sample group (NPS positive, NPS negative, Healthy Volunteer) (Fig. 1b; Supplementary Fig. S2).

Pre-heating of seven saliva samples that consistently returned false negative results after spiking with heat-inactivated virus showed marked improvement—four samples returned a positive result (Fig. 2a, black triangles)—suggesting that some inhibitory components in the saliva could be disrupted or degraded. However, the remaining three samples showed resistance to the treatment, and it was unclear why the long heating step already included in the covidSHIELD method was not sufficient for inactivation of the interferants. Sample dilution was unable to produce correct results for false negative saliva samples (NPS positive, saliva RT-PCR negative) (Fig. 2b, grey circle and square), though this method did remedy false negative results from spiked healthy volunteer samples.

While the utility of saliva as a diagnostic specimen cannot be disputed^15^, and even considering the documented reduction in detection sensitivity relative to NPS samples^16^, our observations provide evidence for a potentially significant issue with a high false negative rate when using minimally processed saliva samples for COVID-19 RT-PCR testing. Indeed, the original manuscript describing the covidSHIELD method^3^ reported 11% false negative results upon initial testing, but 100% sensitivity upon retesting; Kandel et al.^17^ reported 38 of 432 samples (8.8%) as invalid upon initial testing, improving to three invalid samples after retesting; and Sahajpal et al.^5^ reported 5% invalid results (though no false negatives).

Our study indicated that valid samples showing expected C_t_ values for the control RNase P sequence could repeatedly result in a false negative SARS-CoV-2 result, suggesting an inherent issue with interferants in some saliva samples. We note that several other factors could lead to variability in saliva samples, such as the timing of sample collection with respect to disease progression, symptoms, and time of day; and the volume of saliva provided. Additionally, the distribution of the virus within the COVID-19 positive NPS samples and matching saliva samples may have differed, as the viral load within different regions of the upper respiratory tract is not necessarily equal or static^18^. We also recognize that disease prevalence in the population at the time of sample collection and our limited number of samples may have contributed to our observations. However, general agreement in the rate of false negative SARS-CoV-2 RT-PCR results across 137 samples from assessment center attendees and healthy volunteers presented here, and seen in other studies, points to unknown factors that produce significant false negative results with a widely used RT-PCR method. These results indicate that while direct use of minimally processed saliva is desirable to increase the simplicity and reduce the cost for COVID-19 testing, the relatively high rate of false negative results may require additional sample processing, such as RNA extraction, to reduce the possibility of COVID-19 positive citizens unknowingly transmitting the virus within the community.

## Supplementary Information

Supplementary Information.

## Data Availability

All data from this study are included in the published article and the Supplementary Information Files.
