# Supplementary material for: Investigation of discordant SARS-CoV-2 RT-PCR results using minimally processed saliva
Source: Sci Rep. 2022 Feb 18;12:2806. doi: 10.1038/s41598-022-06642-5 (PMC8857222; doi:10.1038/s41598-022-06642-5)
Supplement: Supplementary file 1 — Supplementary Information. [file 41598_2022_6642_MOESM1_ESM.docx]

**Investigation of discordant SARS-CoV-2 RT-PCR results using minimally processed saliva**

**Dawn White^1^, Jimmy Gu^2^, Catherine-Jean Steinberg^3^, Deborah Yamamura^4,5^, Bruno J. Salena^3^, Cynthia Balion^5^, Carlos D.M. Filipe,^6^ Alfredo Capretta^1^, Yingfu Li^1,2,4,7*^, John D. Brennan^1*^**

**Supplementary Information**

**Calibration curve generation**

The 2019-nCoV_N_Positive Control plasmid (ATCC) was used to prepare a 490-base N gene RNA transcript for the generation of an RT-PCR calibration curve representative of the reagents and instrument used: Power SYBR® Green RNA-to-C_T_™ 1-step Kit (ThermoFisher Scientific); CDC N1 forward and reverse primers (IDT); and Biorad CFX96 Touch Real Time PCR system (Biorad). First, a 512-base pair N gene DNA amplicon was created using a forward primer incorporating the T7 RNA polymerase binding site (bold, underlined) (5’–AAATA**TAATACGACTCACTATAG**GGATGTCTGATAATGGACCCCAAAATCAG–3’) and reverse primer (5’–CCTTGAGGAAGTTGTAGCACGATTG–3’) in a standard PCR reaction using 200,000 copies of the control plasmid (2.5 U Biotools DNA polymerase in supplied 1x buffer with 200 nM dNTPs and 100 nM each forward and reverse primers). The amplicon was gel purified (0.8% agarose gel; New England Biolabs Monarch DNA Gel Extraction Kit) and used to generate the 490-base N gene RNA transcript (25 U Invitrogen T7 RNA polymerase in supplied 1x buffer with 30 ng of the N gene DNA amplicon, 250 µM NTPs, 5 mM DTT, 1 U ThermoFisher DNaseI). The N gene RNA transcript was gel purified (6% polyacrylamide/7 M urea; crush and elute extraction^1^) and quantified using the NanoQuant plate accessory of the Tecan SPARK. The IDT PrimerQuest tool was used to designed primers from RT-PCR (forward: 5’-GAAGCTGGACTTCCCTATG-3’; reverse: 5’-CAGCATTGTTAGCAGGATTG-3’). A dilution series of the N gene RNA transcript from 10 pg to 0.01 fg (corresponding to 10^8^ to 10^2^ gc/mL of full length viral genomic RNA) was used to generate a calibration curve (Figure S1, IVT RNA).

A calibration curve was also generated using genomic RNA from 2019 Novel Coronavirus (Isolate USA-WA/2020) purchased from the ATCC. The dilution series from 95 pg to 9.5 fg (corresponding to 10^6^ to 10^2^ gc/mL) overlapped well with the first calibration curve, solidifying confidence in our use of laboratory-based test (Figure S1, ATCC vRNA).

Calibration curves were then generated using serially diluted heat-inactivated virus directly into the RT-PCR reaction mix, where the dilution series was created in water, 100% pooled or 50% pooled human saliva (Innovative Research). Purchased pooled saliva was chosen as the “perfect” saliva sample as it was collected using the passive drool technique without stimulation or coughing and was clear and watery. These calibration curves better represented the samples that would be tested (Figure S1B).

**a**

**b**

**Figure S1**. Calibration curves. (**a**) Calibration curves using In vitro translated (IVT) RNA (R^2^ = 0.9989) and purified genomic viral RNA from ATCC (R^2^ = 0.9841). Samples tested at least in triplicate. (**b**) Calibration curves with heat-inactivated virus in water (R^2^ = 0.9872), 100% pooled saliva (R^2^ = 0.9998) and 50% pooled saliva (R^2^ = 0.9572) used directly in the RT-PCR reaction. Samples tested at least in triplicate.

**Limit of detection**

The limit of detection of the system (LoD) was determined using the dilution by thirds method starting at 10^4^ gc/mL followed by three serial dilutions in water and 50% pooled saliva. Eight replicates of each dilution were processed. The LoD was determined to be 10^4^ gc/mL as the first dilution resulted in at least one negative RT-PCR result (no C_t_ value).

Additionally, of the 50 verified COVID-19 negative saliva samples, eight produced a false positive result, with C_t_ values ranging from 36.12 – 38.89 (average of 37.20 ± 1.04); none of the 23 healthy volunteer saliva samples produced a false positive result. Together, the negative saliva samples gave an 11% false positive rate when C_t_ >36. Therefore, a C_t_ value of >36 was designated as the cut-off value for positivity.

**Control samples for RT-PCR**

One ng of viral genomic RNA extracted from a full 250 µL aliquot of heat-inactivated 2019 Novel Coronavirus (Isolate USA-WA/2020) (from ATCC, through Cedarlane) using the MagMAX Viral/Pathogen Nucleic Acid Isolation Kit (from Applied Biosystems through Fisher Scientific) was included as a positive control for every RT-PCR experiment. All potential samples available for the study were tested with RNase P as the internal human control for the method (CDC RNase P forward and reverse primers from IDT) with an overall average C_t_ value of 27.4 ± 2.2 (C_t_ value range of 22.4 - 34.1). Samples with negative RNase P RT-PCR results were excluded from further investigation. A no template control was included in every RT-PCR experiment, where water was added to the RT-PCR master mix instead of sample.

**Supplementary Table S1:** C_t_ values for human internal control RNase P for all saliva samples used in this study.

| **NPS positive sample** | **C_t_ value** | **NPS negative sample** | **C_t_ value** | **NPS negative sample** | **C_t_ value** | **Healthy volunteer sample** | **C_t_ value** |
| --- | --- | --- | --- | --- | --- | --- | --- |
| 1 | 23.85 | 1 | 30.25 | 26 | 31.03 | 1 | 35.21 |
| 2 | 25.68 | 2 | 27.36 | 27 | 27.04 | 2 | 28.25 |
| 3 | 22.89 | 3 | 26.72 | 28 | 29.11 | 3 | 30.32 |
| 4 | 26.93 | 4 | 28.49 | 29 | 29.11 | 4 | 34.45 |
| 5 | 25.26 | 5 | 27.32 | 30 | 33.31 | 5 | 26.04 |
| 6 | 23.05 | 6 | 26.15 | 31 | 28.69 | 6 | 30.56 |
| 7 | 28.88 | 7 | 30.48 | 32 | 33.55 | 7 | 30.86 |
| 8 | 25.75 | 8 | 27.42 | 33 | 31.02 | 8 | 32.61 |
| 9  10 | 24.74 | 9  10 | 30.10 | 34 | 27.08 | 9  10 | 33.95 |
| 10 | 24.98 | 10 | 28.41 | 35 | 31.99 | 10 | 29.21 |
| 11 | 23.85 | 11 | 30.15 | 36 | 28.90 | 11 | 31.00 |
| 12 | 24.13 | 12 | 28.41 | 37 | 29.55 | 12 | 28.67 |
| 13 | 27.12 | 13 | 27.43 | 38 | 29.76 | 13 | 35.51 |
| 14 | 24.57 | 14 | 32.06 | 39 | 29.91 | 14 | 34.99 |
| 15 | 24.25 | 15 | 28.37 | 40 | 27.17 | 15 | 34.60 |
| 16 | 26.90 | 16 | 31.96 | 41 | 29.45 | 16 | 25.64 |
| 17 | 24.40 | 17 | 30.95 | 42 | 24.99 | 17 | 26.15 |
| 18 | 25.07 | 18 | 33.64 | 43 | 33.74 | 18 | 29.39 |
| 19 | 25.10 | 19 | 25.87 | 44 | 29.34 | 19 | 25.28 |
| 20 | 23.87 | 20 | 27.65 | 45 | 28.46 | 20 | 31.85 |
| 21 | 24.15 | 21 | 29.18 | 46 | 28.17 | 21 | 25.36 |
| 22 | 26.05 | 22 | 27.49 | 47 | 29.47 | 22 | 24.80 |
|  |  | 23 | 29.02 | 48 | 27.36 | 23 | 25.16 |
|  |  | 24 | 30.55 | 49 | 31.13 |  |  |
|  |  | 25 | 22.36 | 50 | 25.10 |  |  |

**Investigation of the addition of Proteinase K to saliva samples**

Given the popular and effective use of Proteinase K during the processing of suspected COVID-19 positive samples to reduce RNA degradation by intrinsic RNases in respiratory fluids, we incorporated this step prior to saliva sample processing. Following the work of Vogels *et al*. (2020), we added the same amount of reagent (Thermo Scientific Proteinase K, Recombinant, PCR grade) to our COVID-19 positive saliva samples then processed the samples as detailed in the methods. We saw no amplification in any of the samples in the presence of Proteinase K, mirroring the result observed by Ranoa *et al*. (2020) during their survey of buffer additives. A control sample prepared with water instead of saliva and processed identically, then spiked with heat-inactivated SARS-CoV-2 also showed no appreciable amplification (C_t_ values of 34.74 and 39.18) indicating that Proteinase K (or a component of the reagent), despite or because of the extended heat treatment within the procedure, had an adverse effect on the downstream RT-PCR reaction using this assay.

**Individual data points for all saliva samples**

The C_t_ value for each saliva sample reported in Figure 1b is represented graphically in Figure S2. All NPS positive samples were confirmed SARS-CoV-2 positive by NPS at the Hamilton Regional Laboratory Medicine Program using a validated laboratory developed RT-PCR method targeting the env and UTR viral RNA regions; the paired saliva samples were tested as received. NPS negative samples were confirmed SARS-CoV-2 negative by NPS as above and were spiked with 10^6^ gc/mL of heat-inactivated SARS-CoV-2 prior to testing. Healthy volunteer samples were spiked with 10^6^ gc/mL of heat-inactivated SARS-CoV-2 prior to testing.

**a**

**b**

**c**

**Figure S2.** Individual C_t_ values for all saliva samples used in this study. (**a**) NPS positive saliva samples tested as received. Circles represent samples with wild-type (Wuhan) SARS-CoV-2; squares represent samples with the Alpha SARS-CoV-2 variant; triangles represent samples with the Gamma SARS-CoV-2 variant. (**b**) NPS negative saliva samples spiked with 10^6^ gc/mL heat-inactivated SARS-CoV-2. (**c**) Healthy volunteer samples spiked with 10^6^ gc/mL heat-inactivated SARS-CoV-2. A C_t_ value of 40 represents a negative result; the black dashed line at C_t_ = 36 represents the cut-off value for our system; the grey dashed line represents the average C_t_ value of all positive samples. Samples were tested at least in duplicate.

**Effect of centrifugation on RT-PCR outcome with saliva samples**

Salivary particulate matter was a cause of higher-than-expected RT-PCR C_t_ values or erroneous false negative results with some samples. Using healthy volunteer saliva samples spiked with 10^6^ gc/mL of heat-inactivated SARS-CoV-2, the negligible difference between allowing a processed saliva sample to settle versus brief centrifugation of the sample was highlighted (Figure S3).

**Figure S3.** Effect of saliva sample settling and centrifugation post-processing. Spiked saliva samples that were allowed to settle (5 minutes; black dots) or were briefly centrifuged (2,000 xg, 2 minutes; grey triangles) after covidSHIELD RT-PCR processing to pellet salivary debris. Samples tested in triplicate.

**Effect of freeze-thaw on RT-PCR performance with saliva samples**

To verify SARS-CoV-2 stability in the saliva samples after a freeze-thaw event, the C_t_ values of samples from each patient group that were tested on separate days were compared. N1 gene amplification was compared for the COVID-19 positive saliva samples (Table S2) and RNase P amplification was compared for the COVID-19 negative saliva samples (Table S3). There was no significant change in the C_t_ values of the samples after one freeze-thaw event for either amplicon.

**Supplementary Table S2:** Comparison of C_t_ values for N1 RT-PCR of COVID-19 positive saliva

samples after one freeze-thaw cycle. ND = Not done.

| **Sample** | **First duplicate** | | **Second duplicate** | | **AVG** | **SD** |
| --- | --- | --- | --- | --- | --- | --- |
| 1 | 27.36 | 27.33 | 28.41 | 28.17 | 27.82 | 0.55 |
| 2 | 33.47 | 31.47 | 34.04 | 34.71 | 33.42 | 1.40 |
| 5 | 31.76 | 33.08 | 35.79 | 34.68 | 33.83 | 1.77 |
| 6 | 33.44 | 33.04 | 35.69 | 35.00 | 34.29 | 1.26 |
| 7 | 27.03 | 27.00 | 26.08 | 26.30 | 26.60 | 0.48 |
| 10 | 34.49 | 35.24 | 34.17 | 33.39 | 34.32 | 0.77 |
| 11 | 30.33 | 30.37 | 30.95 | 30.88 | 30.63 | 0.33 |
| 12 | 31.91 | 31.99 | 32.14 | 32.08 | 32.03 | 0.10 |
| 13 | 28.59 | 27.22 | 28.31 | ND | 28.04 | 0.72 |
| 14 | 34.08 | 34.33 | 34.90 | 34.24 | 34.39 | 0.36 |
| 15 | 32.25 | 31.30 | 31.75 | 31.84 | 31.79 | 0.39 |
| 19 | 31.97 | 31.31 | 33.27 | 34.73 | 32.82 | 1.51 |

**Supplementary Table S3:** Comparison of C_t_ values for RNase P RT-PCR of COVID-19 negative saliva samples after one freeze-thaw cycle.

| **Sample** | **First run** | **Second run** | | **AVG** | **SD** |
| --- | --- | --- | --- | --- | --- |
| 2 | 27.36 | 26.34 | 26.51 | 26.74 | 0.55 |
| 4 | 28.49 | 27.42 | 27.99 | 27.97 | 0.54 |
| 6 | 26.15 | 27.05 | 27.67 | 26.96 | 0.76 |
| 7 | 30.38 | 30.59 | 30.58 | 30.52 | 0.12 |
| 8 | 27.42 | 28.58 | 33.07 | 29.69 | 2.98 |
| 23 | 29.02 | 25.67 | 26.26 | 26.98 | 1.79 |
| 26 | 31.03 | 26.92 | 27.26 | 28.40 | 2.28 |
| 31 | 28.69 | 30.15 | 27.77 | 28.87 | 1.20 |
| 33 | 31.02 | 27.07 | 26.80 | 28.30 | 2.36 |
| 38 | 29.76 | 27.82 | 30.63 | 29.40 | 1.44 |
| 44 | 29.34 | 27.65 | NA | 28.50 | 1.20 |
| 45 | 28.46 | 27.35 | 27.40 | 27.74 | 0.63 |

**Effect of saliva sample dilution on RT-PCR C_t_ values**

The variation in RT-PCR C_t_ values seen with dilution of false negative saliva samples (Figure 2b) was further investigated with five saliva samples from each sample group (COVID-19 positive, COVID-19 negative, and healthy volunteers) after dilution to 10% with sterile RNase-free water. Table S4 shows the original RNase P C_t_ value, the C_t_ value after dilution, and the change in C_t_ value (ΔC_t_).

**Supplementary Table S4:** Change in C_t_ values (ΔC_t_) for RNase P RT-PCR of COVID-19 positive, COVID-19 negative, and healthy volunteer saliva samples after dilution to 10 per cent.

| **Sample** | **COVID-19 Status** | **Original RNase P C_t_ value** | **C_t_ value of 10-fold dilution** | **ΔC_t_ value** |
| --- | --- | --- | --- | --- |
| 1 | Positive | 23.85 | 35.02 | 11.17 |
| 3 | Positive | 22.89 | 33.18 | 10.29 |
| 6 | Positive | 23.05 | 29.24 | 6.19 |
| 12 | Positive | 24.13 | 30.18 | 6.05 |
| 18 | Positive | 25.07 | 34.59 | 9.52 |
| 2 | Negative | 27.36 | 30.17 | 2.81 |
| 3 | Negative | 26.72 | 31.93 | 5.21 |
| 19 | Negative | 25.87 | 29.04 | 3.17 |
| 34 | Negative | 27.08 | 33.76 | 6.68 |
| 50 | Negative | 25.10 | 29.48 | 4.38 |
| 2 | Healthy Volunteer | 28.25 | 31.05 | 2.80 |
| 3 | Healthy Volunteer | 30.32 | 31.03 | 0.71 |
| 5 | Healthy Volunteer | 26.04 | 30.69 | 4.65 |
| 9 | Healthy Volunteer | 33.95 | 29.39 | -4.56 |
| 12 | Healthy Volunteer | 28.67 | 29.54 | 0.87 |

This data suggested that, regardless of the amplicon, the ΔC_t_ remained unpredictable (a 10% reduction in target input should result in a ΔC_t_ of ~3.3 in a perfect system^2^), likely due to the viscous and heterogeneous nature of the saliva sample. An interesting observation was the difference in the average ΔC_t_ between the sample groups: COVID-19 positive saliva samples had the highest ΔC_t_ at 8.64 ± 2.38; COVID-19 negative saliva samples showed half the ΔC_t_ at 4.45 ± 1.57; and saliva samples from healthy volunteers showed the smallest, and closest to expected, ΔC_t_ at 2.26 ± 1.86 (excluding the outlier that showed improved amplification with dilution).

**References**

1. Petrov, A., Wu, T., Puglisi, E. V. & Puglisi, J. D. RNA purification by preparative polyacrylamide gel electrophoresis. *Methods Enzymol*. **530,** 315-30 (2013).
2. Real-Time Applications Guide Bulletin 5279. Bio-Rad Laboratories, Inc (2006).
